# The protective effects of dehydrocostus lactone against TNF-α-induced degeneration of extracellular matrix (ECM) in SW1353 cells

**DOI:** 10.18632/aging.103657

**Published:** 2020-09-14

**Authors:** Lin Wang, Min Yang, Chi Zhang, Fei Huang

**Affiliations:** 1 Department of Orthopaedics, Yijishan Hospital of Wannan Medical College, Wuhu 241000, Anhui Province, China; 2 Department of Orthopaedics, The Fourth Affiliated Hospital of Anhui Medical University, Hefei 230012, Anhui Province, China

**Keywords:** osteoarthritis, dehydrocostus lactone, oxidative stress, pro-inflammatory cytokines, NF-κB

## Abstract

Osteoarthritis is a common joint disease that disrupts the lives of millions of people worldwide. To date, a safe and reliable treatment has not yet been announced. Excessive production of pro-inflammatory cytokines such as TNF-α plays an important role in the pathological development of OA. Dehydrocostus lactone (DHC) is a kind of sesquiterpene isolated from medicinal plants that has been demonstrated to play a protective role in inflammation and tumor formation. However, the effects of DHC in OA hasn’t been reported before. In the present study, we investigated the antioxidant and protective effects of DHC in human chondrocytes against insult from tumor necrosis factor-α (TNF-α). We found that DHC inhibited oxidative stress by suppressing the production of reactive oxygen species (ROS) from TNF-α stimulation. Furthermore, DHC decreased the expression of pro-inflammatory cytokines induced by TNF-α, such as interleukin-1β (IL-1β) and interleukin-6 (IL-6). Importantly, DHC prevented the degradation of type II collagen and aggrecan, which are the main components of the extracellular matrix (ECM), by inhibiting the overexpression of matrix metalloproteinases (MMPs) and a disintegrin and metalloproteinase with a thrombospondin type 1 motif (ADAMTS) induced by TNF-α. Mechanistically, DHC ameliorated the inflammatory response and degeneration of the articular extracellular matrix (ECM) by suppressing nuclear factor-κB (NF-κB) activation. Our results reveal that DHC possesses a beneficial effect against TNF-α-mediated insult in human chondrocytes, implying a potential role for DHC in the treatment of osteoarthritis (OA).

## INTRODUCTION

Osteoarthritis (OA) is considered the world’s most common debilitating skeletal disorder, affecting millions of people around the globe [1]. OA causes chronic inflammation, pain, and disability, especially in elderly patients. While this disease can affect all joints, it is most commonly observed in the knees, hands, and hips [2, 3]. However, the pathogenesis of OA is complicated as it comprises numerous risk factors, including joint injury, obesity, excessive mechanical stress, and aging [4–6]. Indeed, as the average age of the global population continues to increase along with the prevalence of obesity and sedentary lifestyles, it can be expected that a similar increase will be seen in the number of patients suffering from OA [7]. To date, the pathophysiology of OA remains poorly understood. In recent years, increasing evidence has demonstrated that excessive degradation of the cartilage extracellular matrix (ECM) plays a central role in the progression and development of OA. The ECM is primarily comprised of type II collagen, a fibrous protein that acts as the structural scaffold of the ECM, and aggrecan, a proteoglycan that provides cartilage with its shock-absorptive characteristics [8]. Researchers have been actively seeking treatments that can prevent excessive degradation of the ECM, which may, therefore, serve as effective therapeutic strategies against OA [9].

The chondrocyte is the only cell type present in articular cartilage. In normal conditions, this unique cell type exists in a quiescent state. Healthy chondrocytes maintain cartilage homeostasis by strictly regulating anabolic and catabolic processes [10]. However, in pathological conditions, chondrocytes are activated to produce proinflammatory cytokines, including tumor necrosis factor α (TNF-α), interleukin (IL)-1β, and IL-6, which play pivotal roles in disease development by promoting the degradation of the ECM [11–13]. Specifically, upregulation of these cytokines significantly increases the expression of matrix-degrading proteins such as matrix metalloproteinases (MMPs) and a disintegrin and metalloproteinase with a thrombospondin type 1 motif (ADAMTS), especially MMP-1, MMP-3, MMP-13, ADAMTS-4, and ADAMTS-5, which contribute to the degradation of type II collagen and aggrecan [14]. In addition, TNF-α stimulation enhances the activity of the nuclear factor-κB (NF-κB) signaling pathway, which forms a positive feedback loop between NF-κB and pro-inflammatory cytokines, resulting in severe ECM degradation [15]. Oxidative stress and excessive production of reactive oxygen species (ROS) have been associated with the pathogenesis of OA through regulating intracellular signaling processes, chondrocyte senescence, and apoptosis, extracellular matrix synthesis and degradation along with synovial inflammation and dysfunction of the subchondral bone [16, 17].

Dehydrocostus lactone (DHC) is a kind of sesquiterpene isolated from medicinal plants such as *Saussurea lappa*, *Inulahelenium L*., and *Magnolia sieboldii* [18]. DHC has been shown to play a protective role against inflammation and tumor formation [19]. DHC is able to prevent osteoclast-related bone loss during osteoclastogenesis by suppressing receptor activator of nuclear factor-κB ligand (RANKL)-induced osteoclast formation and osteoclast marker gene expression [20]. Another study reported that DHC inhibited the expression of inducible nitric oxide synthase enzyme and the production of NO and TNF-α in lipopolysaccharide (LPS)-activated RAW 264.7 cells [21]. Importantly, these effects of DHC are associated with inhibition of the NF-κB signaling pathway [22, 23], which is a major regulator in various inflammatory diseases. Previous research has also demonstrated an ability of DHC to inhibit the release of MMPs, including MMP-2 and MMP-9 [24]. In light of this, we hypothesized that DHC may provide protection against ECM degradation in OA. However, there have been few studies regarding the potential of DHC in the treatment of OA. In this study, we aimed to investigate whether DHC could protect the articular ECM from degradation induced by TNF-α.

## RESULTS

### DHC ameliorated TNF-α-induced oxidative stress

**Figure 1:**
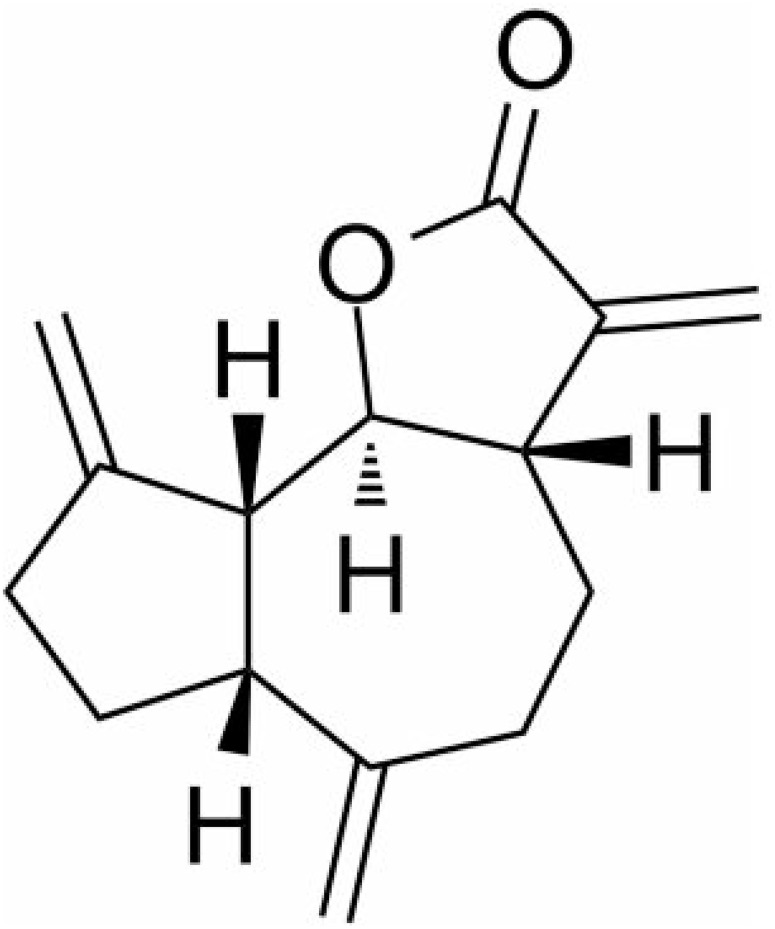
**Molecular structure of DHC.**

**Figure 2:**
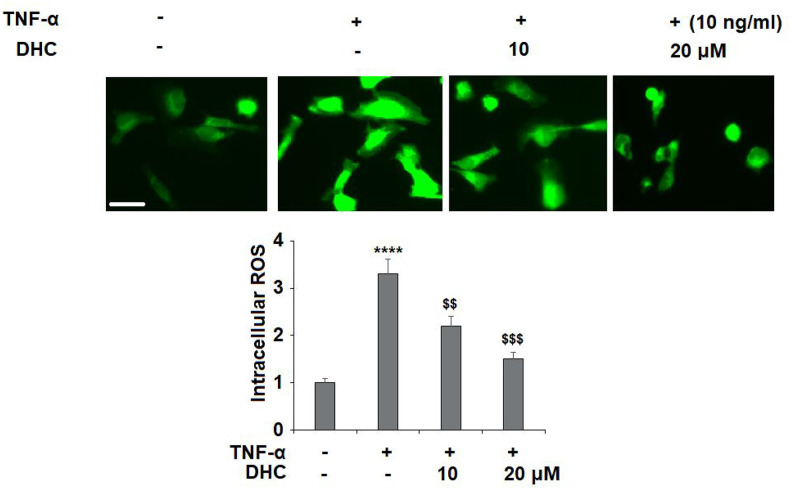
**DHC ameliorated TNF-α-induced oxidative stress in chondrocytes.** Cells were stimulated with TNF-α (10 ng/ml) in the presence or absence of 10 and 20 μM for 24 h. Intracellular ROS was measured by DCFH-DA staining. Scale bar, 100 μm (****, P<0.0001 vs. vehicle group; $$, $$$$, P<0.01, 0.0001 vs. TNF-α treatment group).

The molecular structure is shown in Figure 1. In order to investigate the effects of DHC on TNF-α-induced oxidative stress, the levels of reactive oxygen species (ROS) were measured. As shown in Figure 2, TNF-α induced a 3.3-fold increase in ROS, which was significantly reduced to 2.2- and 1.5-fold by 10 and 20 μM DHC.

### DHC prevented TNF-α-induced expression and secretion of IL-6 and IL-1β

**Figure 3:**
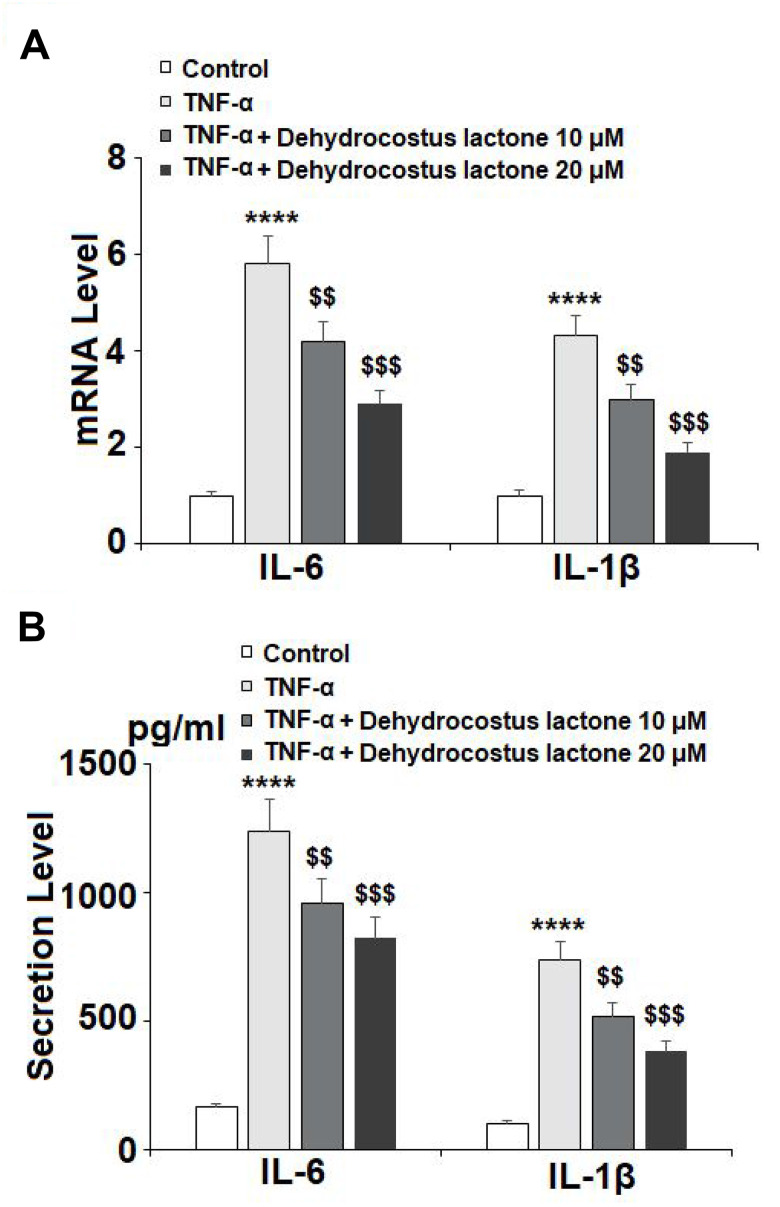
**DHC prevented TNF-α-induced expression and secretion of IL-6 and IL-1β.** Cells were stimulated with TNF-α (10 ng/ml) in the presence or absence of 10 and 20 μM for 24 h. (**A**). mRNA of IL-6 and IL-1β as measured by real-time PCR; (**B**). Secretion of IL-6 and IL-1β as measured by ELISA (****, P<0.0001 vs. vehicle group; $$, $$$$, P<0.01, 0.0001 vs. TNF-α treatment group).

To confirm the inhibitory effects of DHC on the release of proinflammatory cytokines, the mRNA and protein levels of IL-6 and IL-1β were measured. The results in Figure 3A show that the mRNA levels of IL-6 and IL-1β were respectively increased to 5.8- and 4.3-fold by exposure to TNF-α alone. However, 10 μM DHC reduced the mRNA levels of these proinflammatory cytokines to only 4.2- and 3.0-fold, which were further decreased to 2.9- and 1.9-fold by 20 μM DHC, respectively. Similarly, TNF-α treatment increased the protein levels of IL-6 and IL-1β from 165.8 and 105.1 pg/ml to 1235.2 and 735.6 pg/ml, which were reduced to 956.7 and 516.8 pg/ml by 10 μM DHC, and to 822.1 and 382.3 pg/ml by 20 μM DHC, respectively (Figure 3B). Thus, DHC exerted a strong inhibitory effect against TNF-α-induced expression of proinflammatory cytokines.

### DHC prevented TNF-α-induced degradation of type II collagen by reducing the expression of MMP-1, MMP-3, and MMP-13 in human SW1353 chondrocytes

**Figure 4:**
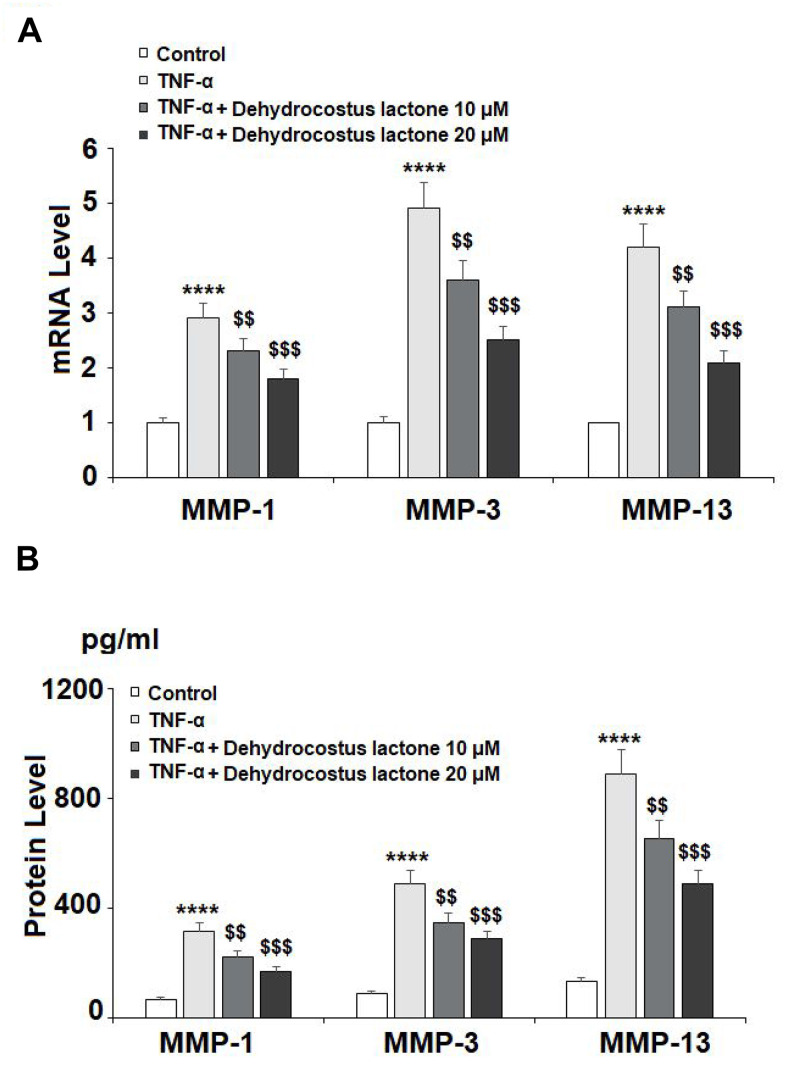
**DHC reduced TNF-α- induced expression of MMP-1, MMP-3, and MMP-13.** Cells were stimulated with TNF-α (10 ng/ml) in the presence or absence of 10 and 20 μM for 24 h. (**A**). mRNA of MMP-1, MMP-3, and MMP-13 as measured by real time PCR; (**B**). Protein of MMP-1, MMP-3, and MMP-13 as measured by ELISA (****, P<0.0001 vs. vehicle group; $$, $$$$, P<0.01, 0.0001 vs. TNF-α treatment group).

**Figure 5:**
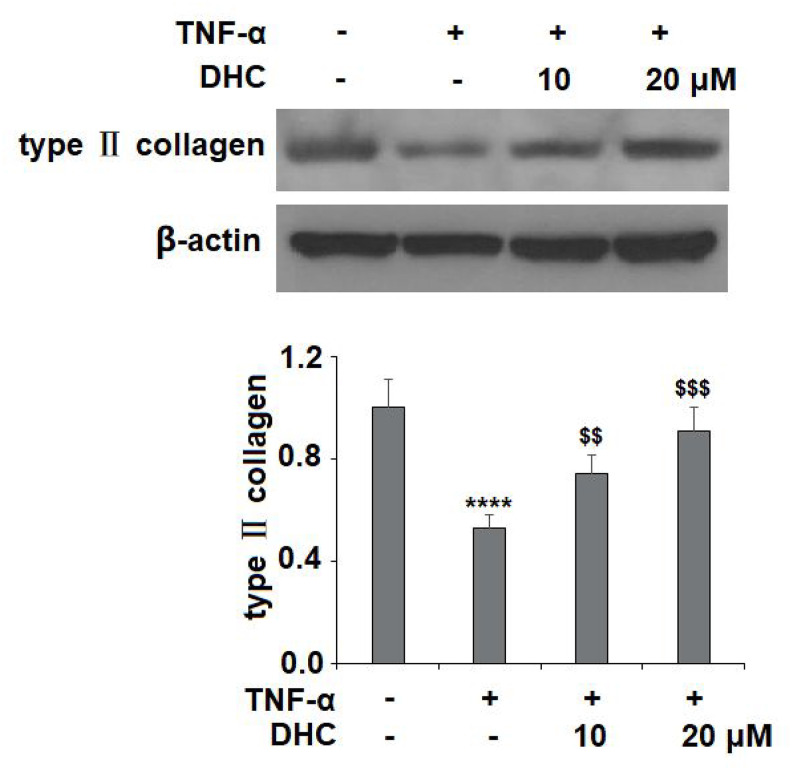
**DHC prevented TNF-α- induced degradation of type II collagen.** Cells were stimulated with TNF-α (10 ng/ml) in the presence or absence of 10 and 20 μM for 24 h. The expression of type II collagen was measured by western blot analysis (****, P<0.0001 vs. vehicle group; $$, $$$$, P<0.01, 0.0001 vs. TNF-α treatment group).

As shown in Figure 4A, TNF-α treatment significantly enhanced the expression of MMP-1, MMP-3, and MMP-13 to 2.9-, 4.9-, and 4.2-fold, respectively, at the mRNA level, while treatment with 10 μM DHC reduced these increases to only 2.3-, 3.6-, and 3.1-fold. Indeed, 20 μM DHC further reduced the mRNA levels of these enzymes to 1.8-, 2.5-, and 2.1-fold. Meanwhile, the results in Figure 4B show that TNF-α treatment stimulated the protein expression of MMP-1, MMP-3, and MMP-13, causing an increase from 69.4, 88.3, and 135.1 pg/ml to 316.7, 486.9, and 886.6 pg/ml, respectively. However, 10 μM DHC reduced the protein levels of these enzymes to 221.2, 345.7, and 653.1 pg/ml, which were further decreased to 168.7, 288.2, and 489.5 pg/ml by 20 μM DHC, respectively. In addition, we investigated the expression of type II collagen. TNF-α decreased the expression of type II collagen to 53%, which was rescued to 74% and 91% by 10 and 20 μM DHC, respectively (Figure 5). Thus, DHC exhibited a protective effect on type II collagen by inhibiting the expression of MMP-1, MMP-3, and MMP-13.

### DHC prevented TNF-α-induced degradation of aggrecan by inhibiting expression of ADAMTS-4 and ADAMTS-5

**Figure 6:**
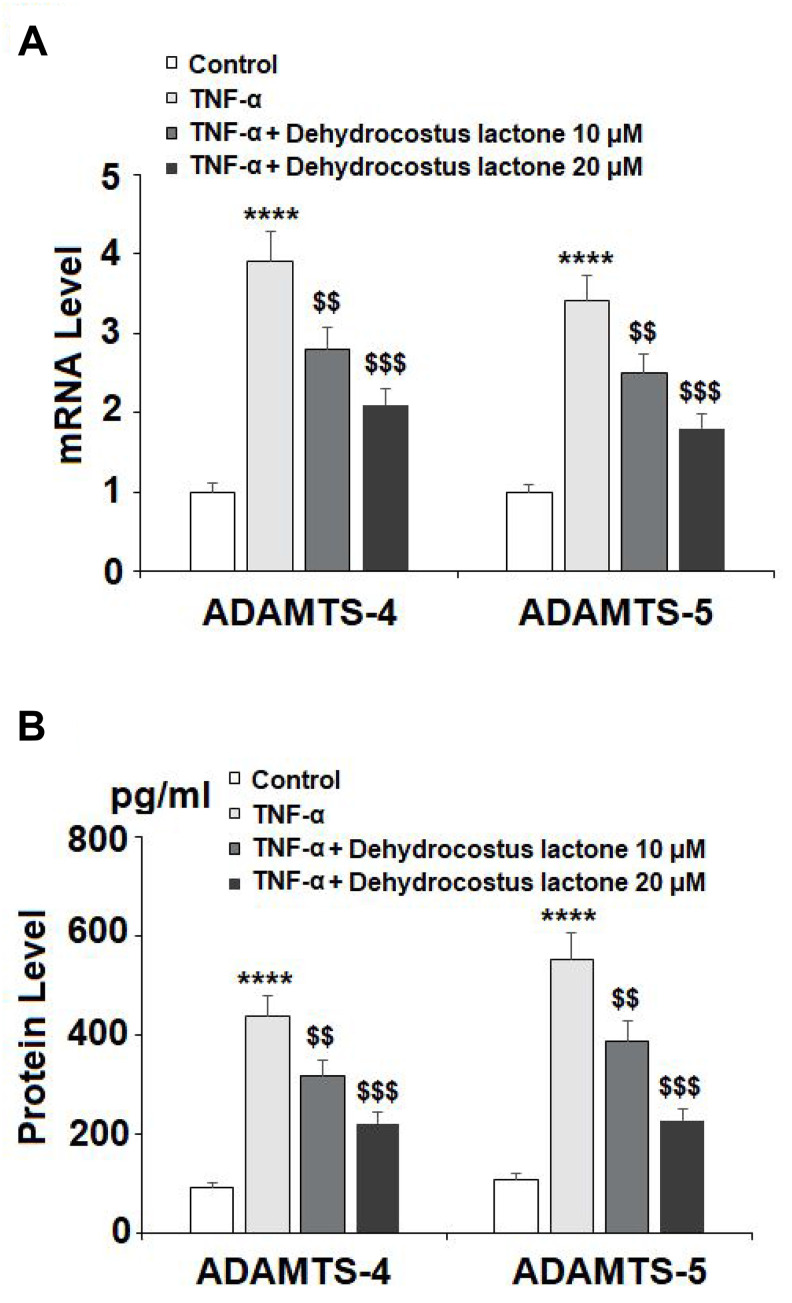
**DHC reduced TNF-α- induced expression of ADAMTS-4 and ADAMTS-5.** Cells were stimulated with TNF-α (10 ng/ml) in the presence or absence of 10 and 20 μM for 24 h. (A). mRNA of ADAMTS-4 and ADAMTS-5 as measured by real-time PCR; (B). Protein of ADAMTS-4 and ADAMTS-5 as measured by ELISA (****, P<0.0001 vs. vehicle group; $$, $$$$, P<0.01, 0.0001 vs. TNF-α treatment group).

**Figure 7:**
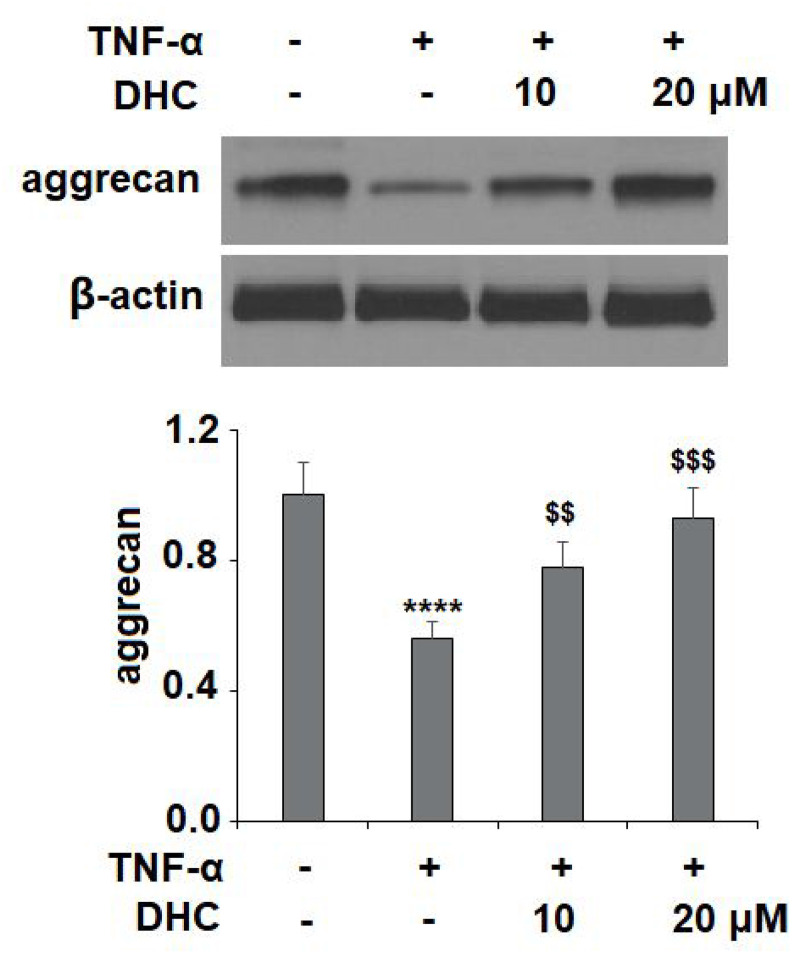
**DHC prevented TNF-α-induced degradation of aggrecan.** Cells were stimulated with TNF-α (10 ng/ml) in the presence or absence of 10 and 20 μM for 24 h. The expression of aggrecan was measured by western blot analysis (****, P<0.0001 vs. vehicle group; $$, $$$$, P<0.01, 0.0001 vs. TNF-α treatment group).

It is well known that ADAMTS-4 and ADAMTS-5 are responsible for the degradation of aggrecan. The results in Figure 6A show that TNF-α treatment increased the mRNA levels of ADAMTS-4 and ADAMTS-5 to 3.9- and 3.4-fold, which were reduced to 2.8- and 2.5-fold by 10 μM DHC, respectively. Concordantly, 20 μM DHC further decreased the mRNA levels of these enzymes to 2.1- and 1.8-fold. The results in Figure 6B show that the protein levels of ADAMTS-4 and ADAMTS-5 were significantly enhanced from 93.6 and 109.4 pg/ml to 436.6 and 551.8 pg/ml, respectively, by exposure to TNF-α alone. However, 10 and 20 μM DHC reduced the protein levels of ADAMTS-4 to 316.7 and 221.5 pg/ml, and ADAMTS-5 to 388.2 and 227.9 pg/ml, respectively. We then measured the expression of aggrecan. TNF-α treatment reduced the expression of aggrecan to 56%, which was reduced to only 78% and 93% by 10 and 20 μM DHC, respectively (Figure 7). These findings demonstrate that DHC protected aggrecan from TNF-α-induced degradation by suppressing the expression of ADAMTS-4 and ADAMTS-5.

### DHC prevented TNF-α-induced activation of NF-κB in human SW1353 chondrocytes

**Figure 8:**
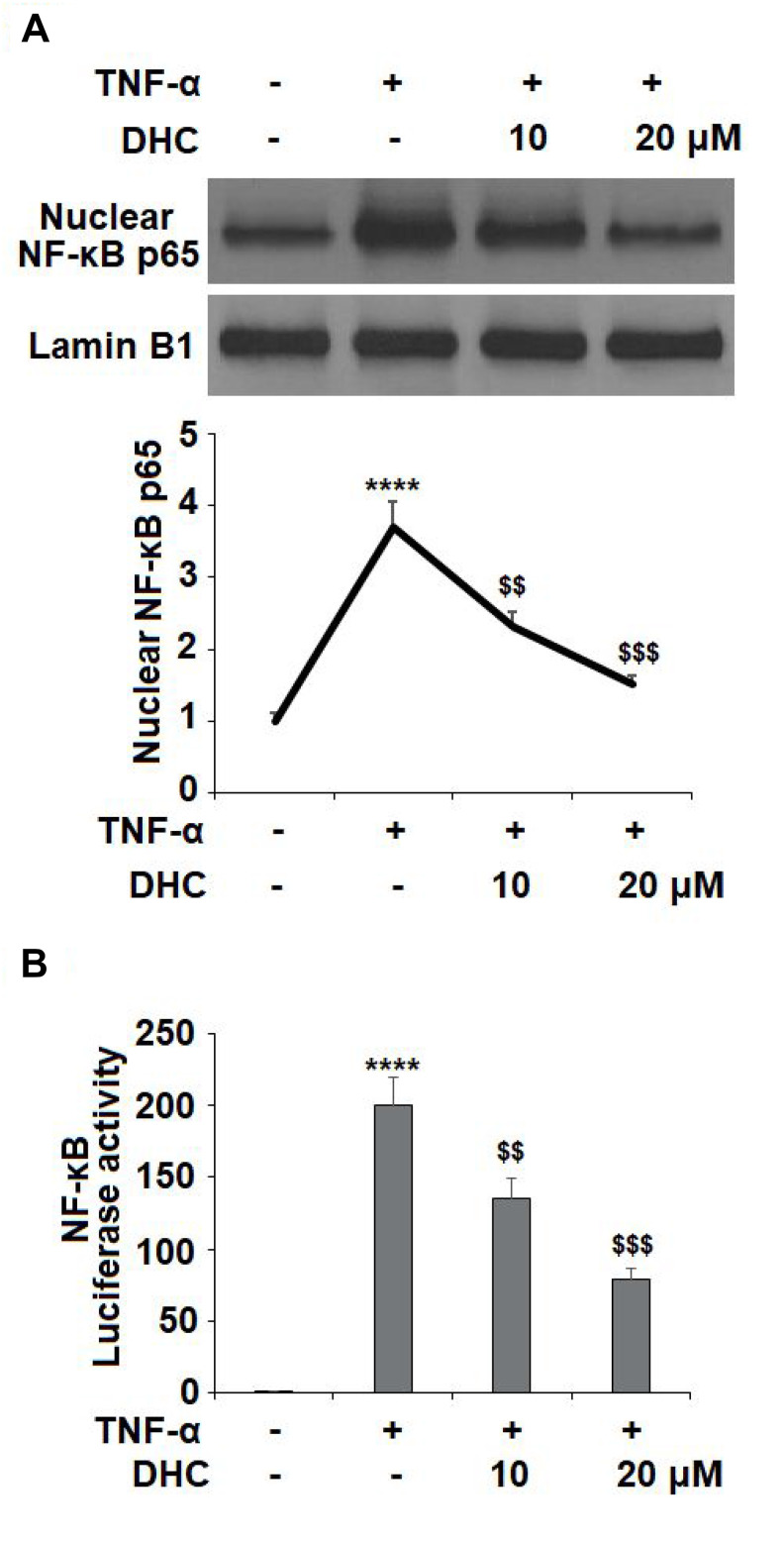
**DHC prevented TNF-α- induced activation of NF-κB.** Cells were stimulated with TNF-α (10 ng/ml) in the presence or absence of 10 and 20 μM for 24 h. (**A**). Nuclear levels of NF-κB p65; (**B**). Luciferase activity of NF-κB (****, P<0.0001 vs. vehicle group; $$, $$$$, P<0.01, 0.0001 vs. TNF-α treatment group).

Activation of NF-κB acts as a master modulator in the development of OA by regulating the expression of various cytokines and enzymes. NF-κB is typically sequestered in the cytoplasm in an inactive state, but upon activation by TNF-α or other inflammatory cytokines, the p65 subunit of NF-κB translocates to the nucleus where it triggers the transcription and activation NF-κB. The results in Figure 8A show that TNF-α stimulation increased the nuclear level of NF-κB p65 to 3.7-fold, which was reduced to 2.3- and 1.5-fold by 10 and 20 μM DHC, respectively. Similarly, the luciferase activity of NF-κB was increased to 199.8-fold by exposure to TNF-α alone. However, the same doses of DHC inhibited the luciferase activity of NF-κB to 135.6- and 78.5- fold (Figure 8B). Thus, DHC likely exerts its anti-inflammatory effect through the inhibition of NF-κB signaling.

## DISCUSSION

OA is a debilitating joint disorder characterized by chronic inflammation, synovial tissue infiltration, and excessive cartilage degradation. It is well-known that in the pathogenesis of OA, oxidative stress resulting from an imbalance in ROS production and SOD activity contributes to chondrocyte dysfunction and apoptosis [25, 26]. In addition, the overproduction of ROS negatively affects the ECM by suppressing the synthesis of type II collagen and aggrecan and enhancing the degeneration of the ECM. ROS have been shown to reduce the production of type II collagen and aggrecan by inhibiting mitochondrial oxidative phosphorylation and adenosine triphosphate formation [27]. Meanwhile, DHC has been shown to inhibit the production of ROS and rescue SOD activity in several *in vitro* models [28–30]. In this study, TNF-α stimulation induced an increase in the production of ROS. As expected, 10 and 20 μM DHC suppressed the production of ROS to near baseline. Thus we determined that DHC exerts a considerable antioxidant effect in TNF-α-stimulated chondrocytes.

The expression of pro-inflammatory cytokines, including TNF-α, IL-1β, and IL-6, is considered to play a vital role in the pathogenesis of OA. Upregulated expression of pro-inflammatory cytokines is associated with chronic inflammation as well as physical symptoms such as reduced articular functionality and pain in the development of OA [31–33]. Importantly, studies have demonstrated that TNF-α and IL-1β are responsible for the degradation of the ECM as they upregulate the expression of MMPs and ADAMTS, thereby triggering the expression of downstream cytokines and pro-inflammatory signaling pathways [34, 35]. DHC has demonstrated an inhibitory effect against TNF-α and IL-1β in both *in vitro* and *in vivo* models [36, 37]. In the present study, our findings indicate that DHC exerts a distinct dose-dependent inhibitory effect against the expression of IL-1β and IL-6 induced by TNF-α in chondrocytes (Figures 3A and 3B).

As a hallmark of OA, the excessive degradation of the ECM might be the most important treatment target of OA therapies. In this study, we demonstrated that TNF-α stimulation significantly increased the expression of MMPs and ADAMTS, especially MMP-1, MMP-3, MMP-13, ADAMTS-4, and ADAMT-5. MMPs are the major hydrolytic enzymes involved in the cleavage of type II collagen [16, 37]. Meanwhile, ADAMTS-4 and ADAMTS-5 are responsible for the degradation of aggrecan [38, 39]. Our results indicate that DHC treatment prevented the degradation of type II collagen by inhibiting the expression of MMP-1, MMP-3, and MMP-13, which were enhanced by TNF-α stimulation. Furthermore, DHC rescued aggrecan from degradation by decreasing the expression of ADAMTS-4 and ADAMTS-5 induced by TNF-α treatment. These findings indicate that DHC exerts a protective effect on the ECM by rescuing type II collagen and aggrecan from degradation induced by TNF-α. Furthermore, DHC regulates the activation of NF-κB signaling.

**Figure 9:**
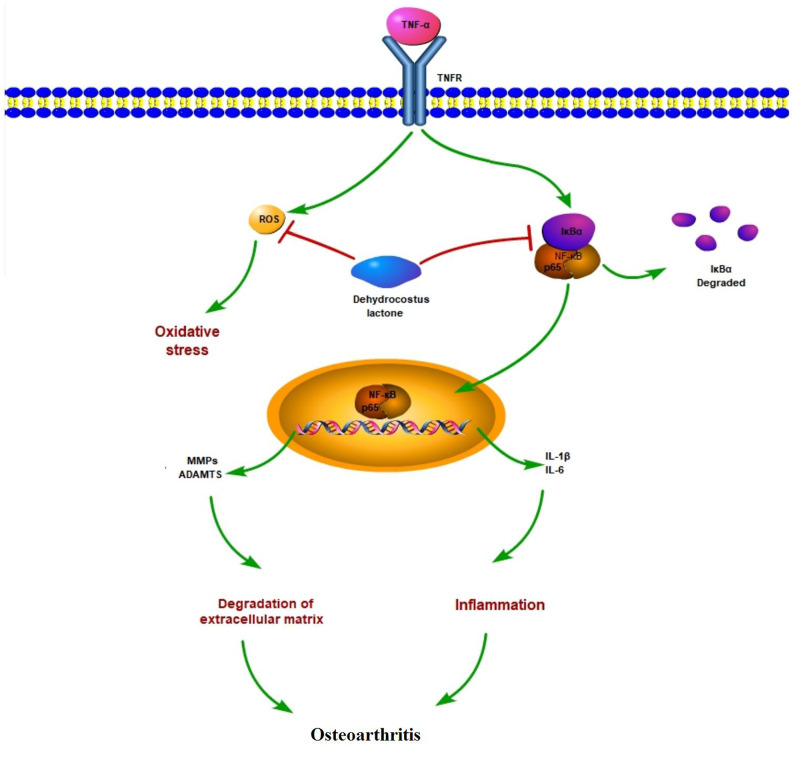
**Graphic summary of the molecular model.**

It is well known that the role of the NF-κB signaling pathway has been widely explored in various diseases and acts as a master regulator of inflammation in the development of OA [40]. The activation of NF-κB induces the expression of a large number of pro-inflammatory cytokines and chemokines that initiate the process of joint destruction. Under normal conditions, NF-κB is sequestered in the cytoplasm by IκBα, which inhibits NF-κB activity. However, when IκBα is phosphorylated, the p65 subunit translocates to the nucleus, where it triggers the activation of NF-κB and, subsequently, a cascade of deleterious effects. Thus, inhibition of NF-κB activation is often considered as a therapeutic target. NF-κB is abnormally activated in OA and as a disease-contributing factor [41]. NF-κB participates in many OA-associated events, including chondrocyte inflammation, chondrocyte proliferation, and synovial inflammation [42]. Thus, NF-κB has been considered as a promising target for the therapeutic intervention of OA. As expected, we found that DHC could suppress the activation of NF-κB by inhibiting the nuclear translocation of p65 induced by TNF-α. This finding is congruent with a recent study in which DHC was shown to inhibit osteoclastogenesis and osteoclast-mediated bone loss by inhibiting the activation of NF- κB via the suppression of IKK/IκBα/p65 signaling [20]. Another contemporary study demonstrated that DHC could inhibit NF-κB activation via modulation of the toll-like receptor (TLR) signaling pathway [43]. To our knowledge, this study is the first to demonstrate the ability of DHC to suppress NF-κB activation in chondrocytes. A graphical representation of a molecular model involved in the current study is shown in Figure 9.

However, the present study has several limitations. Firstly, we only examined the effects of DHC in chondrocytes *in vitro*. While previous research has demonstrated the safety of DHC *in vivo* [44], further investigation is required to expand our understanding of how treatment with DHC affects other tissues and cell types. Our future studies will include animal models of OA to elucidate whether DHC can slow OA progression in organisms. Secondly, we stimulated SW1353 chondrocytes with TNF-α to simulate the pathological process driving OA development *in vitro*. While TNF-α is recognized as a major driver of OA pathogenesis [45], OA is a complicated disease with a multitude of factors contributing to its progression. Therefore, additional studies are required to understand the effects of DHC on other cellular signaling pathways.

Together, the findings of the present study provide a basis for further research on the potential of DHC as a therapeutic option to treat or prevent the development of OA.

## MATERIALS AND METHODS

### Cell culture and treatment

SW1353 human chondrocytes purchased from the Shanghai Institute of Cell Biology, the Chinese Academy of Sciences (Shanghai, China) were used in our experiments. Briefly, the cells were cultured in a humidified incubator (5% CO_2_/37 °C) in L-15 medium (Sigma-Aldrich, USA) containing 10% fetal bovine serum (FBS) (Gibco, USA) and 1% antibiotics (100 U/ml penicillin/ 100 μg/ml streptomycin) (Sigma-Aldrich, USA). The medium was changed every 2-3 days. When the cells had reached full confluence, they were stimulated with TNF-α (10 ng/ml) [46] (R&D Systems, USA) in the presence or absence of 10 and 20 μM DHC (Target Molecule Corp., China) for 24 h.

### Real-time PCR

Real-time PCR analysis was performed to detect the mRNA expression of the target genes using SYBR Green Master Mix (Thermo Fisher Scientific, USA). Briefly, total RNA was isolated from chondrocytes using Qiazol reagent (Qiagen, USA). The quality and concentration of the extracted RNA were determined using a NanoDrop microvolume spectrophotometer. Then, 2 μg of the isolated RNA was reverse transcribed into cDNA using an iScript cDNA Synthesis kit (Bio-Rad, USA). Real-time PCR was performed using glyceraldehyde-3-phosphate dehydrogenase (GAPDH) as an internal housekeeping gene. The 2^-ΔΔCT^ threshold cycle method was used to normalize the expression levels of the target genes to GAPDH. The following primers were listed in Table 1.

**Table 1:** Primer sequences.

**Target gene**	**Upstream Sequence (5′-3′)**	**Downstream Sequence (5′-3′)**
MMP-1	5′-AGCTAGCTCAGGATGACATTGATG -3′;	5′- GCCGATGGGCTGGACAG -3′;
MMP-3	5′-TTAAAATAAAACTGCTTTT-3′;	5′- AACTGGAGCATTTTTT-3′;
MMP-13	5′-AGGAGCATGGCGACTTCTAC-3′;	5′-TAAAAACAGCTCCGCATCAA-3′;
ADAMTS-4	5′-ACACTGAGGACTGCCCAAC-3′;	5′-GGTGAGTTTGCACTGGTCCT-3′;
ADAMTS-5	TCT 5′-GCAGAACATCGACCAACTCTACTC-3′;	5′ - CCAGCAATGCCCACCGAAC -3′;
IL-6	5′-GGTACATCCTCGACGGCATCT-3′;	5′-GTGCCTCTTTGCTGCTTTCAC-3′;
IL-1β	5′- AAGCTGATGGCCCTAAACAG -3′;	5′-AGGTGCATCGTGCACATAAG -3′;
GAPDH	5′- ACTGGCGTCTTCACCACCAT-3′;	5′- AAGGCCATGCCAGTGAGCTT-3′.

### Western blot analysis

Western blot analysis was used to determine the protein expression of the target genes. Chondrocytes were seeded into 6-well plates and then subjected to the indicated treatment. Then, the total protein was isolated from the cells using cell lysis buffer (Cell Signaling Technology, USA) with protease and phosphatase inhibitors (buffer: PMSF: PhosSTOP = 100:1:1) (Cell Signaling Technology, USA). Next, samples of 20 μg protein were separated using 10% sodium dodecyl sulphate-polyacrylamide gel electrophoresis (SDS-PAGE) and then transferred onto polyvinylidene fluoride (PVDF) membranes (Bio-Rad, USA). The membranes were blocked with 5% non-fat milk at room temperature (RT) to bind the non-specific sites and then probed with primary antibodies overnight at 4 °C. The membranes were washed 3 times with tris buffered saline tween (TBST) and incubated overnight with horseradish peroxidase (HRP)-conjugated secondary antibodies. Antibodies were diluted in TBST. An enhanced chemiluminescence kit (Thermo Fisher Scientific, USA) was used to develop the blots, and the fluorescent signal was detected using fluorescence microscopy. The following antibodies were used in this study: mouse monoclonal antibody (mAb) against type II collagen (1:1000, #MAB8887, Chemicon, USA); mouse mAb against aggrecan (1:1000, #ab3778, Abcam, USA); anti-rabbit IgG, HRP-linked secondary antibody (1:3000, #7074, Cell Signaling Technology, USA); anti-mouse IgG, HRP-linked antibody (1:3000, #7076, Cell Signaling Technology, USA); mouse mAb against β-actin (1:10000, #3700, Cell Signaling Technology, USA).

### Enzyme linked immunosorbent assay (ELISA)

After the treatment described above, the culture media was collected, and chondrocyte cell lysates were obtained using RIPA buffer. Enzyme-linked immunosorbent assay (ELISA) kits from R&D Systems were used to determine the protein concentrations of IL-1β, IL-6, MMP-1, MMP-3, MMP-13, ADAMTS-4, and ADAMTS-5. The following ELISA kits were used: Human IL-1β Quantikine ELISA Kit (#DLB50); Human IL-6 Quantikine ELISA Kit (#D6050); Human MMP-1 DuoSet ELISA Kit (DY901B); Human MMP-3 DuoSet ELISA Kit (#DY513-05); Human MMP-13 DuoSet ELISA (#DY511); Human ADAMTS4 DuoSet ELISA (#DY4307-05); Human ADAMTS5 DuoSet ELISA (#DY2198-05).

### 2’,7’-dichlorofluorescin diacetate (DCFH-DA) staining

The cells were subjected to the indicated treatment, and then the levels of intracellular ROS were assessed using the dye DCFH-DA (Sigma-Aldrich, USA). After the cells were washed 3 times with PBS buffer, they were stained with 5 μM DCFH-DA for 30 min. After 3 washes, an IBE2000 inverted fluorescence microscope (Zeiss, Germany) was used to visualize the fluorescence signals with excitation at 510 nm and emission: 580 nm. The level of intracellular ROS is based on the average fluorescence intensity.

### NF-κB luciferase activity

To determine the activity of NF-κB, we employed an NF-κB luciferase promoter vector (Clontech, USA). Briefly, the cells were cotransfected with NF-κB promoter paired with a firefly luciferase vector using Lipofectamine 2000 reagent (Thermo Fisher Scientific, USA). The cells were subjected to the treatment described above and then lysed with cell lysis buffer. Renilla luciferase was used an internal control. The luciferase activity was measured using a dual-luciferase reporter assay system kit from Promega, USA.

### Statistical analysis

The results of our experiments are presented as means ± standard error of measurement (S.E.M.). All experiments were repeated in triplicate. Statistical analysis of the results was performed using SPSS software (Version 21.0). One-way analysis of variance (ANOVA) followed by the post-hoc Bonferroni test was used to determine the statistical significance of differences. P<0.05 was considered to represent a statistically significant value.
